# On system-spanning demixing properties of cell polarization

**DOI:** 10.1371/journal.pone.0218328

**Published:** 2019-06-21

**Authors:** Fabian Bergmann, Walter Zimmermann

**Affiliations:** Theoretische Physik I, Universität Bayreuth, 95440 Bayreuth, Germany; Universitat Pompeu Fabra, SPAIN

## Abstract

A number of mathematical models have been suggested to describe cell polarization in eukaryotic cells. One class of models takes into account that certain proteins are conserved on the time scale of cell polarization and may switch between a fast and a slow diffusing state. We raise the question whether models sharing this design feature can be condensed into one system-spanning model. We show exemplarily for the mass-conserved reaction-diffusion model of Otsuji et al. (Otsuji M et al. (2007) PLoS Comput Biol 3(6):e108) that cell polarization can be classified as active phase separation. This includes a fundamental connection between a number of non-equilibrium demixing phenomena such as cell polarization to phase separation. As shown recently, generic properties of active phase separation close to its onset are described by the Cahn-Hilliard model. By a systematic perturbation analysis we directly map the basic cell polarization model to the universal Cahn-Hilliard model. Comparing the numerical solutions of the polarization model and the Cahn-Hilliard equation also provides the parameter range where the basic cell polarization model behaves like other systems showing active phase separation. Polarization models of the active phase separation type cover essential properties of cell polarization, e.g. the adaptability of cell polarity to the length of growing cells. Our approach highlights how basic principles of pattern formation theory allow the identification of common basic properties in different models for cell polarization.

## Introduction

Cell polarization is one of many fascinating self-organized patterns in living systems that has simultaneously an important functionality [1–8]. During the polarization of living cells certain proteins are enriched in the front and back half of the cell [9–22]. This breaks the symmetry of the cell and defines a unique axis. Polarization of cells is therefore crucial for cell locomotion, the orientation of cell divisions in tissues and the formation of organized multicellular structures [9]. But since cell polarization is this crucial for the reliability of biological processes, we address the question whether these different kinds of polarization follow a similar and robust syntax (at least in certain parameter ranges). All examples differ in the participating proteins, the type of interactions and their trigger mechanisms. However, they also have several features in common. Cell polarization occurs on time scales of minutes. On these time scales degradation or de-novo production of proteins is negligible. Therefore the total amount of the respective proteins is conserved inside the cell [13–20, 22]. Simulations of numerous different mathematical models of such mass-conserved systems often provide similar results, e.g. showing a transition to a globally similar polarization state. Finally, cell polarization with mass-conserved reaction-diffusion models resembles very much a demixing process. However, all cell polarization processes involve the consumption of some kind of mostly chemical energy to perform directed movement, rendering them non-equilibrium phenomena. For this class of non-equilibrium transitions the notion active phase separation was coined recently [23]. This class unites non-equilibrium demixing phenomena that are induced by so-called type II instabilities [3] and fulfill a global (mass) conservation constraint. Apart from cell polarization, examples for active phase separation range from clustering of chemotactically communicating cells [24–28], to self-propelled particles [29–33], patterning in active matter models [34], mixtures of particles with different models of ion-channel densities [35] or mussels in ecology [36]. We showed in Ref. [23] that near onset of active phase separation the Cahn-Hilliard (CH) model [37, 38] is the universal order parameter equation. We demonstrated a related perturbative reduction scheme for two very elementary models. Here we show that mass-conserved reaction diffusion systems with two involved fields belong to the class of systems showing active phase separation. We thereby use the established model for cell polarization introduced by Otsuji et al. [14] as a seminal example. We derive the universal CH equation directly from the established cell polarization model by applying the perturbational expansion introduced in Ref. [23], while also showing their applicability of in the general case. Since the reduction method applies especially close to the onset of cell polarization, we compare the polarization model with the reduced model in this neighborhood. We thereby consider both stationary solutions as well as the dynamics of the cell polarization model and its reduction.

Considering cell polarization as a realization of a universal equation–at least close to its onset—opens a new route to explain why cell polarization is often very similar across many different systems. We thereby especially show and discuss how the dynamics of the Cahn-Hilliard equation offers an explanation for similarities in mass-conserved reaction-diffusion systems. Our results may therefore help to identify and understand collective and universal features such as spontaneous polarization, adaptability to the cell length and robustness of the polarization pattern.

## Results and discussion

### Cell polarization model

A class of models for cell polarization share the unifying feature of fast and slow diffusing forms of the same type of signal molecules [14–18, 39]. These are for instance different forms of GTPases: an active, membrane-bound and slowly diffusing form $\tilde{u}$, and an inactive and fast diffusing counterpart $\tilde{v}$. The overall number of signal molecules with two different states is conserved on the time scale of cell polarization. In this temporal regime the two states of signal molecules are described by two coupled reaction-diffusion equations for $\tilde{u},\tilde{v}$ of the following form:

$$\begin{array}{c} \partial_{t}\tilde{u}=D_{u}\nabla^{2}\tilde{u}+f\left(\tilde{u},\tilde{v}\right)\;, \end{array}$$ (1)

$$\begin{array}{c} \partial_{t}\tilde{v}=D_{v}\nabla^{2}\tilde{v}-f\left(\tilde{u},\tilde{v}\right)\;. \end{array}$$ (2)

The symmetrical reaction term $f(\tilde{u},\tilde{v})$ with two different signs in both equations reflects the overall conservation of the signal molecules.

Here we exemplarily analyze the model of Otsuji et al. [14, 19], with the reaction term

$$\begin{array}{c} f\left(\tilde{u},\tilde{v}\right)=a_{1}(\tilde{v}-\frac{\tilde{u}+\tilde{v}}{{\left(a_{2}\left(\tilde{u}+\tilde{v}\right)+1\right)}^{2}}). \end{array}$$ (3)

Note we extend this analysis to arbitrary reaction terms *f*(*u*, *v*) in S1 Appendix. For simplicity reasons we restrict ourselves to one spatial dimension for most parts of the work. In this case the global conservation condition reads

$$\begin{array}{c} N=\frac{1}{L}\int_{0}^{L}\left[\tilde{u}\left(x\right)+\tilde{v}\left(x\right)\right]dx\;. \end{array}$$ (4)

The coupled equations in Eqs (1) and (2) have the homogeneous basic solution

$$\begin{array}{c} u_{h}=\frac{a_{2}N^{2}\left(a_{2}N+2\right)}{{\left(a_{2}N+1\right)}^{2}}\;, \end{array}$$ (5)

$$\begin{array}{c} v_{h}=\frac{N}{{\left(a_{2}N+1\right)}^{2}}\;. \end{array}$$ (6)

### Onset of cell polarization

We first separate the homogeneous parts *u*_*h*_ and *v*_*h*_ from the inhomogeneous parts *u* and *v* with

$$\begin{array}{c} \tilde{u}=u_{h}+u\;, \end{array}$$ (7)

$$\begin{array}{c} \tilde{v}=v_{h}+v\;. \end{array}$$ (8)

At first we assume small inhomogeneous perturbations |*u*|, |*v*| ≪ *u*_*h*_, *v*_*h*_ with respect to the basic state. This allows for a linearization of the basic equations (Eqs (1) and (2)) with respect to small perturbations *u*, *v* leading to two coupled equations:

$$\begin{array}{c} \partial_{t}u=D_{u}\partial_{x}^{2}u+f_{u}u+f_{v}v\;, \end{array}$$ (9)

$$\begin{array}{c} \partial_{t}v=D_{v}\partial_{x}^{2}v-f_{u}u-f_{v}v\;, \end{array}$$ (10)

with

$$\begin{array}{c} f_{u}=\partial_{u}f{|}_{u=u_{h},v=v_{h}}=\frac{a_{1}\left(a_{2}N-1\right)}{{\left(a_{2}N+1\right)}^{3}}\;, \end{array}$$ (11)

$$\begin{array}{c} f_{v}=\partial_{v}f{|}_{u=u_{h},v=v_{h}}=\frac{a_{1}a_{2}N\left(a_{2}^{2}N^{2}+3a_{2}N+4\right)}{{\left(a_{2}N+1\right)}^{3}}\;. \end{array}$$ (12)

The two coupled equations in Eqs (9) and (10) are solved by

$$\begin{array}{c} u,v=\bar{u},\bar{v}\;e^{\sigma t+iqx}\;. \end{array}$$ (13)

The two resulting linear equations for $\bar{u}$ and $\bar{v}$ have a solubility condition leading to a quadratic polynomial for the growth rate *σ*:

$$\begin{array}{cc} \sigma^{2} & +[\left(D_{u}+D_{v}\right)q^{2}+f_{v}-f_{u}]\sigma \\ & +D_{u}D_{v}q^{4}+\left(D_{u}f_{v}-D_{v}f_{u}\right)q^{2}=0\;. \end{array}$$ (14)

For positive values of the parameter *a*_1_ in Eq (3) one has *f*_*v*_ > *f*_*u*_. In this case an expansion of the root *σ*_+_(*q*) up to the order *q*^4^ gives

$$\begin{array}{c} \sigma_{+}=G_{2}q^{2}-G_{4}q^{4}O\left(q^{6}\right)\;, \end{array}$$ (15)

with

$$\begin{array}{c} G_{2}=\frac{D_{v}f_{u}-D_{u}f_{v}}{f_{v}-f_{u}}\;,\;G_{4}=\frac{{\left(D_{u}-D_{v}\right)}^{2}f_{u}f_{v}}{{\left(f_{v}-f_{u}\right)}^{3}}\;. \end{array}$$ (16)

The growth rate *σ*_+_(*q*) becomes positive in a finite range of *q*, if *G*_2_ > 0. Choosing *D*_*v*_ as the control parameter, the homogeneous state loses stability and *G*_2_ becomes positive for

$$\begin{array}{c} D_{v}>D_{v}^{c}=D_{u}\frac{f_{v}}{f_{u}}. \end{array}$$ (17)

This critical value $D_{v}^{c}$ marks the onset of cell polarization. We introduce a small quantity *ε* in order to parameterize the control parameter *D*_*v*_ near its critical value $D_{v}^{c}$:

$$\begin{array}{c} D_{v}=D_{v}^{c}\left(1+\varepsilon \right)\;. \end{array}$$ (18)

**Fig 1:**
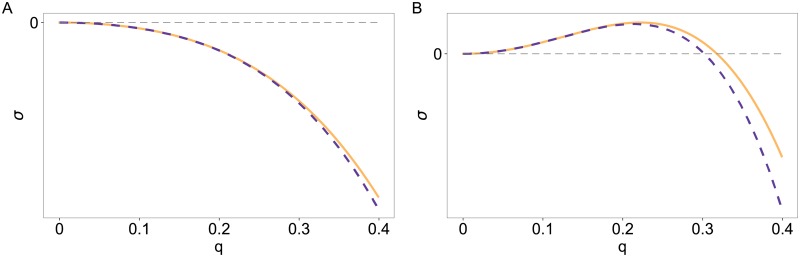
Growth rate *σ*_+_ as a function of the wavenumber *q*. Comparison between the full solution in Eq (14) (solid line) and its Taylor expansion up to the order *q*^4^ given by Eq (15) (dashed line) at the critical point *ε* = 0 (A) and slightly above *ε* = 0.1 (B) (for *a*_2_ = 2).

At the critical point (*ε* = *ε*_*c*_ = 0) the maximum of the growth rate is at *q* = 0 (see Fig 1A). Raising the control parameter *ε* shifts this maximum to finite values of *q*. For *ε* > 0 there is a range [0 < |*q*| < *q*_right_] with a positive growth rate *σ* > 0 (see Fig 1B). In contrast to classical Turing patterns, this range of positive growth rate extends down to *q* = 0, which is a signature of the overall conservation of the two densities $\tilde{u}$ and $\tilde{v}$. Fig 1 additionally shows a comparison of the full dispersion relation (14) and its approximation up to order *q*^4^ in Eq (15).

### Derivation of the generic Cahn-Hilliard model

The two concentration fields *u*(*x*, *t*) and *v*(*x*, *t*) are coupled by the conservation law in Eq (4). We show that near the onset of cell polarization the dynamics of both fields can be described by only one order parameter field. The dynamical equation for this field is the famous Cahn-Hilliard (CH) model for demixing phenomena at and far from thermal equilibrium (see e.g. [23] and references therein), as we derive in the following. Note, that this is in contrast to a so-called Galerkin method made under the assumption of spatially periodic solutions of the model of Otsuji et al, cf. [19], which we do not find in a wide parameter range around the onset of cell polarization.

We introduce the vector fields **w** = (*u*, *v*) and **N** = (*f*(*u*, *v*), − *f*(*u*, *v*)) and rewrite the two coupled Eqs (1) and (2) in terms of the two vector fields in a compact form:

$$\begin{array}{c} \partial_{t}w=Lw+N\;. \end{array}$$ (19)

Considering again the growth rate in Eq (15), the upper limit of the *q*-range with positive *σ* is $q_{max}\propto \sqrt{\varepsilon }$. Therefore, the inhomogeneous parts *u*(*x*, *t*) and *v*(*x*, *t*), cf. Eqs (7) and (8), are slowly varying functions in space. This suggests the introduction of a new spatial scale with $X=\sqrt{\varepsilon }x$ and two slow time scales *T* = *ε*^2^*t* and *T*_3_ = *ε*^3/2^*t*. These scales lead to the following replacements of the spatial and temporal derivatives:

$$\begin{array}{c} \partial_{x}\to \sqrt{\varepsilon }\partial_{X}\;, \end{array}$$ (20)

$$\begin{array}{c} \partial_{t}\to \varepsilon^{3/2}\partial_{T_{3}}+\varepsilon^{2}\partial_{T}\;. \end{array}$$ (21)

Note that the introduction of two different time scales is necessary to fulfill the solvability condition in the hierarchy of equations following below in Eqs (24), (25), (26), (27) and (28) (see also supplement S1 Appendix for additional information). Here we consider the basic equations (see Eqs (1) and (2)) in the range of small modulations $u,v∼\sqrt{\varepsilon }$ of the average concentrations *u*_*h*_ and *v*_*h*_. Accordingly we expand the field **w** with respect to the small parameter *ε* as follows:

$$\begin{array}{c} w=\varepsilon^{1/2}w_{1}+\varepsilon w_{2}+\varepsilon^{3/2}w_{3}+\varepsilon^{2}w_{4}+\varepsilon^{5/2}w_{5}+...\;, \end{array}$$ (22)

leading to

$$\begin{array}{c} N=\varepsilon N_{2}+\varepsilon^{3/2}N_{3}+\varepsilon^{2}N_{4}+\varepsilon^{5/2}N_{5}+.... \end{array}$$ (23)

Inserting this expansion of the field **w** and the derivatives in Eqs (20) and (21) results in the following *ε*-hierarchy of equations:

$$\begin{array}{cc} \varepsilon^{1/2} & :L_{0}w_{1}=0\;, \end{array}$$ (24)

$$\begin{array}{cc} \varepsilon \;\;\;\; & :L_{0}w_{2}=-N_{2}\;, \end{array}$$ (25)

$$\begin{array}{cc} \varepsilon^{3/2} & :L_{0}w_{3}=-L_{1}\partial_{X}^{2}w_{1}-N_{3}\;, \end{array}$$ (26)

$$\begin{array}{cc} \varepsilon^{2\;}\;\; & :L_{0}w_{4}=\partial_{T_{3}}w_{1}-L_{1}\partial_{X}^{2}w_{2}-N_{4}\;, \end{array}$$ (27)

$$\begin{array}{cc} \varepsilon^{5/2} & :L_{0}w_{5}=\partial_{T_{3}}w_{2}+\partial_{T}w_{1}-L_{1}\partial_{X}^{2}w_{3}-L_{2}\partial_{X}^{2}w_{1}-N_{5}\;. \end{array}$$ (28)

Solving the eigenvalue equation in $O(\sqrt{\varepsilon })$ leads to

$$\begin{array}{c} u_{1}\left(x,t\right)=f_{v}\tilde{A}\left(x,t\right)\;,\;v_{1}\left(x,t\right)=-f_{u}\tilde{A}\left(x,t\right)\;. \end{array}$$ (29)

This means the two fields *u*_1_(*x*, *t*) and *v*_1_(*x*, *t*) are proportional to each other whereby the eigenvector (*f*_*v*_, −*f*_*u*_)^*T*^ of $L_{0}$ is the proportionality factor. With this starting point the hierarchy of Eqs (24), (25), (26), (27) and (28) is solved successively as described in more detail in the supplemental part S1 Appendix. The solubility conditions at the orders *ε*^2^ and *ε*^5/2^ provide expressions for $\partial_{T_{3}}\tilde{A}$ and $\partial_{T}\tilde{A}$. After reconstituting the original scalings *x* and *t* in Eqs (20) and (21) via $\partial_{t}\tilde{A}=\partial_{T_{3}}\tilde{A}+\partial_{T}\tilde{A}$ we obtain an equation for $A\left(x,t\right)=\sqrt{\varepsilon }\tilde{A}$. This equation has the form of the Cahn-Hilliard equation [37, 38, 40] with an additional quadratic term:

$$\begin{array}{c} \partial_{t}A=-\partial_{x}^{2}[\gamma_{1}\varepsilon A+\gamma_{2}\partial_{x}^{2}A-\gamma_{3}A^{2}-\gamma_{4}A^{3}]\;. \end{array}$$ (30)

The parameters *γ*_*i*_ are determined in terms of the parameters of the starting model in Eqs (1), (2) and (4) as follows:

$$\begin{array}{c} \gamma_{1}=\frac{D_{u}f_{v}}{a_{1}}=D_{u}\frac{a_{2}N\left(a_{2}^{2}N^{2}+3a_{2}N+4\right)}{{\left(a_{2}N+1\right)}^{3}}\;, \end{array}$$ (31)

$$\begin{array}{c} \gamma_{2}=\frac{D_{u}^{2}f_{v}}{a_{1}f_{u}}=D_{u}^{2}\frac{a_{2}N\left(a_{2}^{2}N^{2}+3a_{2}N+4\right)}{a_{1}\left(a_{2}N-1\right)}\;, \end{array}$$ (32)

$$\begin{array}{c} \gamma_{3}=\frac{D_{u}}{f_{u}}\frac{a_{1}^{2}a_{2}\left(a_{2}N-2\right)}{{\left(a_{2}N+1\right)}^{4}}=D_{u}\frac{a_{1}a_{2}\left(a_{2}N-2\right)}{1-a_{2}^{2}N^{2}}\;, \end{array}$$ (33)

$$\begin{array}{c} \gamma_{4}=\frac{D_{u}}{f_{u}}\frac{a_{2}^{2}a_{1}^{3}\left(3-a_{2}N\right)}{{\left(a_{2}N+1\right)}^{5}}=\frac{D_{u}a_{1}^{2}a_{2}^{2}\left(3-a_{2}N\right)}{\left(a_{2}N+1\right)\left(a_{2}^{2}N^{2}-1\right)}\;. \end{array}$$ (34)

Among the coefficients, *γ*_1_ is always positive. To make the linear part of Eq (30) (considered in Fourier space) capture the approximate dispersion relation given by Eq (15) *γ*_2_ has to be positive, i.e. *a*_2_ *N* > 1. The nonlinear coefficient *γ*_4_ is positive in the range 1 < *a*_2_ *N* < 3. In this range the CH model has a cubic limitation term. If *γ*_4_ is negative there is no limiting nonlinearity, i.e. the reduction is no longer valid and would require going to higher orders of *ε* [23]. In this work we focus on the range of *γ*_4_ > 0 where cell polarization close to its onset belongs to the universal class of active phase separation [23]. Within this range the coefficient *γ*_3_ changes its sign at *a*_2_ *N* = 2. For *γ*_3_ = 0 cell polarization is symmetric close to its onset because the CH model in Eq (30) has a ±*A*-symmetry in this case. In this instance the transition to cell polarization takes place continuously or supercritically in the language of pattern formation [2]. The reduction of the basic model in Eqs (1) and (2) to the CH model thereby allows the important distinction between the parameter ranges where cell polarization takes place continuously or discontinuously. Eq (30) can also be represented by a variational derivative of a related functional

$$\begin{array}{c} \partial_{t}A=\frac{\partial^{2}}{\partial x^{2}}\frac{\delta F}{\delta A}\;, \end{array}$$ (35)

with

$$\begin{array}{c} F=\int dx(-\frac{\gamma_{1}\varepsilon }{2}A^{2}+\frac{\gamma_{2}}{2}{\left(\partial_{x}A\right)}^{2}+\frac{\gamma_{3}}{3}A^{3}+\frac{\gamma_{4}}{4}A^{4})\;. \end{array}$$ (36)

It is a surprising result that the order parameter field *A*(*x*, *t*) follows the potential dynamics according to Eq (35), because the basic model in Eqs (1) and (2) cannot be derived from a functional even in the range of small *ε* for which the CH model was derived. However, this phenomenon is not exclusive to cell polarization. The same relaxation dynamics are also found in other demixing systems showing active phase separation [23]. Moreover, the envelope of spatially periodic patterns in non-equilibrium systems, including spatially periodic Turing patterns, also follow potential dynamics while the dissipative starting equations do not (see e.g. [2, 3] and references therein).

These relaxational dynamics of the solution *A* of Eq (30) are helpful for further analysis. For instance, Eq (36) allows to estimate the magnitude of piecewise constant solutions with *A* ≠ 0. These correspond to the minimum of the functional in Eq (36) with respect to *A*. For further details of related analytical considerations we refer to part S2 Appendix of the supplementary information. The constant plateau values of opposite signs are

$$\begin{array}{c} A_{\pm }=\frac{-\gamma_{3}\pm \sqrt{3\gamma_{3}^{2}+9\varepsilon \gamma_{1}\gamma_{4}}}{3\gamma_{4}}\;. \end{array}$$ (37)

In the symmetric case, *γ*_3_ = 0, the Cahn-Hilliard model in Eq (30) has a well known domain wall solution in long systems [38, 40]:

$$\begin{array}{c} A(x)=F\tanh(\frac{x}{\xi_{0}})\;, \end{array}$$ (38)

$$\begin{array}{c} \text{with}\;\xi_{0}=\sqrt{\frac{2\gamma_{2}}{\gamma_{1}\varepsilon }}=\sqrt{\frac{2D_{u}}{\varepsilon f_{u}}}\;,\;F=\pm \sqrt{\frac{\gamma_{1}\varepsilon }{\gamma_{4}}}\;. \end{array}$$ (39)

The coherence length *ξ*_0_ is a measure for the width of the domain wall, the transition range between the positive and negative plateau values of the hyperbolic tangent. The expression for *ξ*_0_ shows that in the context of cell polarization the width of the transition range depends directly on the diffusion constant of membrane-bound state *u* and the distance from the onset of cell polarization. This is an important insight found via the reduction to the CH model in Eq (38).

### Comparison of solutions of the basic and the Cahn-Hilliard model

In this section we determine numerically the steady state solutions of the basic equations for cell polarization in Eqs (1) and (2) and the CH model in Eq (30) in a finite one-dimensional domain of length *L*. Additional informations about the simulations can be found in the materials and methods section. By comparing the steady state solutions of both equations we determine in which parameter range the solutions agree qualitatively or even quantitatively. We focus our simulations on the parameter range with a positive coefficient *γ*_4_ > 0, where cell polarization is limited by a cubic nonlinearity. In the following analysis we therefore keep the parameters *D*_*u*_ = 0.1, *a*_1_ = 3 and *N* = 1 fixed, while *D*_*v*_ and *a*_2_ will be varied.

#### Comparison of steady state solutions in the symmetric case *γ*_3_ = 0

**Fig 2:**
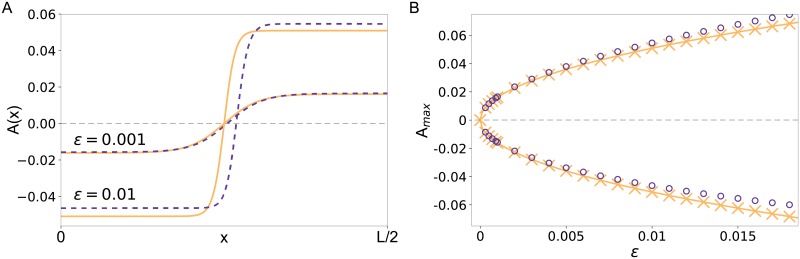
Comparison of steady state solutions of the polarization model and its corresponding CH model in the symmetric case. (A) Steady state profiles for *a*_2_ = 2 and two values of *ε* = 0.001 and *ε* = 0.01 for the polarization model in Eqs (1) and (2) with $A\left(x\right)=\left(v_{h}-\tilde{v}\left(x\right)\right)/f_{u}$ (dashed lines) and for the solution *A*(*x*) of the CH model in Eq (30) (solid lines). (B) Plateau values of the steady state profiles shown in (A) as function of *ε* for the basic polarization model (circles) and the CH model (crosses). The solid line shows an analytical approximation for the plateau values in the CH model given by Eq (37).

For *γ*_3_ = 0 the reduced CH model is ±*A*-symmetric. This is illustrated by the numerical solutions *A*(*x*) of the CH model, in Fig 2A (solid lines) for the two control parameter values *ε* = 0.001, 0.01. Due to the ±-symmetry the solutions of the CH model above the onset of cell polarization show a plateau with increased and a plateau with decreased concentration, each covering exactly half of the system. Increasing the control parameter *ε* leads to increased plateau values as well as more step-like profiles. This trend is also indicated by the coherence length *ξ*_0_ in Eq (39), which decreases with increasing *ε*. For *ε* = 0.001, the cell polarization in the full model is also perfectly symmetric (see Fig 2A, dashed orange line). Additionally the approximation via the CH model matches the results of the full model almost perfectly. Increasing *ε* to *ε* = 0.01 leads to slightly asymmetric polarization in the full model, which can be identified by the off-center shift of the zero-crossing as well as the different magnitude of the plateau values. Since the CH model (for *a*_2_ = 2) is ±-symmetric irrespective of *ε*, the asymmetry in the full model leads to slight deviations between the two.

This means, in the basic model the ±-symmetry is broken with an increasing distance *ε* from the onset of cell polarization. For the derivation of the CH model only contributions up to cubic order in *u* and *v* were taken into account. However, an expansion of the denominator of the function *f*(*u*, *v*) includes also higher terms, such as *u*^4^, *v*^4^, which break the ±-symmetry with increasing *ε*.

To quantify this symmetry-breaking effect and estimate a validity range of our reduction for *γ*_3_ = 0, we also compare the plateau values as a function of *ε* in Fig 2B. Additionally, we approximate the plateau values analytically from the CH model assuming a two plateau solution as in Eq (37). For small values of *ε*, the simulation results of the polarization and the reduced model as well as the analytical solution match almost perfectly. For larger control parameter values, the system-specific ‘dialects’ like the increasing asymmetry begin to play a role which leads to deviations between the full model and the CH equation. Moreover, Fig 2 shows that the transition from the homogenous state to the phase-separated state is smooth, i.e. it occurs in a supercritical bifurcation in the parameter range predicted by our perturbation expansion.

#### Comparison of steady state solutions in the asymmetric case *γ*_3_ ≠ 0

**Fig 3:**
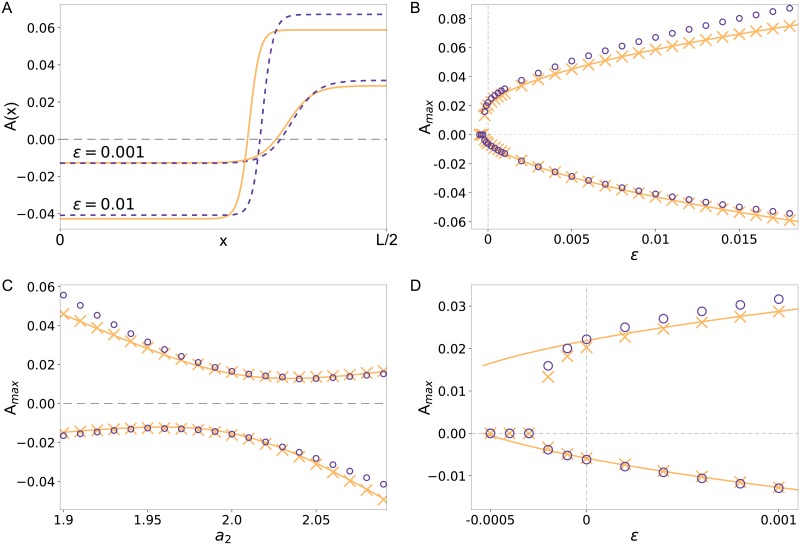
Comparison of steady state solutions of the polarization model and its corresponding CH model in the asymmetric case. (A) Steady state profiles for *a*_2_ *N* = 1.95 and two values of *ε* = 0.001 and *ε* = 0.01 for both the basic polarization model in Eqs (1) and (2) with $A\left(x\right)=\left(v_{h}-\tilde{v}\left(x\right)\right)/f_{u}$ (dashed lines) and the solution *A*(*x*) of the CH model in Eq (30) (solid lines). (B), (D) Plateau values of the steady state profiles shown in (A) from simulations of the basic polarization model (purple circles) and the reduced CH model (yellow crosses) as a function of *ε*. The yellow solid line is an analytical approximation for the plateau values of the CH model given by Eq (37). (D) is a close-up around *ε* = 0 that shows the subcriticality of the bifurcation. (C) Plateau values of the steady state profiles for simulations of the polarization model (purple circles) and the reduced CH model (yellow crosses) as a function of the asymmetry parameter *a*_2_. The yellow line depicts again an approximation via Eq (37).

For *γ*_3_ ≠ 0 the ±-symmetry is already broken immediately at onset. Fig 3A therefore shows that the phase with increased concentration takes a smaller fraction of the system than the phase with decreased concentration or vice versa. For small control parameter values the full polarization model and its corresponding CH equation are in good agreement. For larger values of *ε* the approximation still provides the correct trends but with less predictive power. Comparing the plateau values in Fig 3B and 3D quantifies this validity range. The deviations in the analytical solution compared to the simulations in the range *ε* < 0 are due to the neglection of the interface energy term in the functional that covers the spatial variations between the plateau values. These become more and more relevant if the control parameter is decreased.

Fig 3D also reveals that the broken symmetry at threshold already changes the character of the onset of cell polarization, i.e. for *a*_2_ ≠ 2 the bifurcation is no longer smooth. Instead the transition of the homogenous basic states to polarization is subcritical (discontinuous). In this case we observe a bistable region where both the polarized and the homogenous state are stable. This also implies a hysteretic behavior if we increase and decrease the control parameter: Starting with a negative *ε*, the basic state *A* = 0 stays stable until *ε* = 0. For larger control parameter values we end up in a polarized state. But starting in the polarized state and decreasing the control parameter, the polarized state remains even in a range of negative values of *ε*.

Fig 3C shows a comparison of the polarization model and the corresponding CH equation as a function of the asymmetry parameter *a*_2_ for fixed control parameter *ε* = 0.001. While for small asymmetry the agreement is almost perfect, in case of more asymmetric polarization the system specific ‘dialects’ come into play.

#### Comparison of dynamics

Apart from the stationary profiles considered in the previews section, we also consider the temporal evolution of the polarization model and its corresponding CH equation. Thereby we distinguish between two different scenarios depending on the system size. To measure the system size we express the wavenumber *q*_*max*_ of the fastest growing mode also in terms of the coherence length *ξ*_0_ (see Eq (39))

$$\begin{array}{c} q_{max}^{2}=\frac{1}{\xi_{0}^{2}}\;. \end{array}$$ (40)

**Fig 4:**
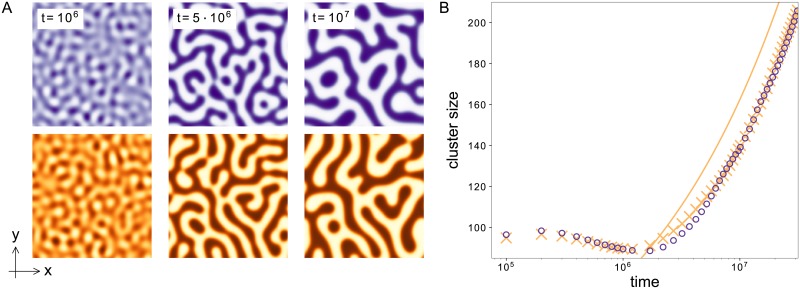
Comparison of the dynamics of the polarization model and its corresponding CH model. (A) 2D snapshots of the full model (upper row, purple) and the corresponding CH model (bottom row, orange) for different times. The Cahn-Hilliard model captures the coarsening dynamics of the full model. (B) Cluster size as a function of time for the polarization model (purple circles) and the corresponding CH model (orange crosses). The solid orange line shows the power law that is expected as a long-term behavior. Parameters: *a*_2_ = 2, *ε* = 0.01.

Note that the length scale *ξ*_0_ of the domain wall between the two polar states also determines the preferred mode during onset of cell polarization. In the case of large system sizes *L* ≫ 2*π*/|*q*_*max*_| we compare in Fig 4A the qualitative dynamics of both the polarization model and its corresponding CH model for a fixed set of parameters in 2D. For a control parameter value above the threshold of pattern formation, the homogenous basic state is unstable. This leads to an initial patterned state whose wavelength is dominated by the fastest growing mode *q*_*max*_. But this patterned state is unstable towards perturbations with a larger wavelength, i.e. the system undergoes a coarsening process. Fig 4A shows 2D snapshots of this coarsening process for different times in a quadratic system of length *L* = 800. At every time step the snapshots of the polarization and the CH model show striking similarities. The figure also depicts the visual similarity between the polarization process modeled here and phase separation of e.g. a liquid-liquid mixture [38]. For a quantitative comparison of the full model and the CH model we compare the dominating wavelength over time. We determine the dominating wavelength (or the cluster size in 2D) using the pair correlation function (see also Methods) on simulation results in 2D. We consider 2D simulations instead of 1D as in the previous section due to the scaling expected from the CH equation. The cluster size in this equation scales logarithmically in 1D, i.e. a comparison is computationally very expensive. In 2D, the cluster size is expected to scale with a power law, which is much more convenient. Fig 4B shows a comparison of the dynamics of the polarization model and its corresponding CH model for the symmetric case and *ε* = 0.01. The CH model captures the dynamics of the full model very well with deviations under 5%. For small times the systems show an interplay between growth of the pattern and the coarsening process which leads to a rather constant cluster size. When the growth process of the amplitudes stops, the dynamics is dominated by coarsening. In this regime the dynamics of both the polarization and the CH model follow the power law $\propto t^{\frac{1}{3}}$. In the language of the original work of Otsuji et al. [14] this coarsening dynamic in 2D corresponds to the observed instability of multi-peak solutions towards a single peak in 1D. The parameters in the original work correspond to a large value of *ε* ≈ 1.8 and a strongly asymmetric cell polarization leading to a more peak-like profile compared to the step-like profiles shown here (see Figs 2A and 3A). Even if a quantitative comparison between the polarization model and the CH equation is no longer valid in this parameter regime similarities in the qualitative dynamical behavior still prevail: Initially a multi-peak solution develops with a wavenumber *q*_*max*_. However this pattern is unstable and evolves or coarsens into a single peak solution. Our analysis shows that this behavior is no surprise for mass-conserved reaction-diffusion systems but instead generic in systems that can be reduced to the CH equation. Nevertheless for biological cell polarization reaching a polar state trough a coarsening process might be too slow. To therefore avoid a coarsening dynamics nature has to tune the system parameters appropriately. If the system length *L* ∼ *O*(2*π*/|*q_m_*|), the short system length suppresses any coarsening. This means if the system parameters are such that the width of the transition *ξ*_0_ is of the order of the system length L, like the extension of a cell, we expect a direct transition to a polar state without a complex intermediate temporal behavior like coarsening regimes. Our analysis allows the identification of the parameters *D*_*u*_, *f*_*u*_ and *ε* where to expect this direct transition to the polar state from an unstable homogenous basic state:

$$\begin{array}{c} L\approx 2\pi \sqrt{\frac{2D_{u}}{\varepsilon f_{u}}}\;. \end{array}$$ (41)

**Fig 5:**
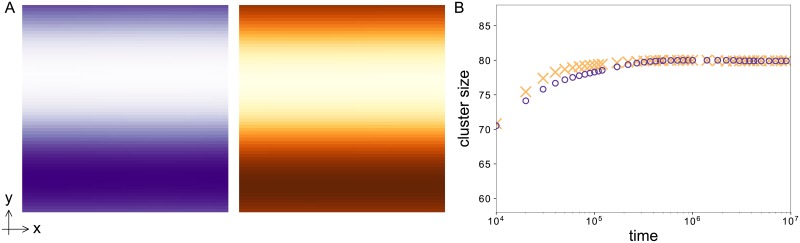
Coarsening is suppressed in small systems. (A) 2D snapshots of the full model (left, purple) and the corresponding CH model (right, orange) for *t* = 5 ⋅ 10^6^ in a small system (*L* = 80). Both simulations show a fully phase separated (one peak) system. (B) Cluster size as a function of time for the polarization model (purple circles) and the corresponding CH model (orange crosses). The cluster size almost immediately goes to the system size *L* = 80. Parameters: *a*_2_ = 2, *ε* = 0.01.

For the chosen parameters, this is the case for *L* ≈ 84. We verify this claim in simulations with a system length *L* = 80 shown in Fig 5. As expected, the average cluster size (see Fig 5B) almost immediately approaches the system size and stays constant from there on. The final state is a fully polar system with one region with increased and one with decreased concentration (see 2D snapshots in Fig 5A). This state corresponds to what is called a single-peak solution in 1D in Ref. [14]. Note, however, that suppressing the coarsening dynamics does not require the system length to fit the condition in Eq (41) perfectly. Since the initially growing wavelength is unstable towards larger wavelengths, the system tends to settle into the largest wavelength, which has also been verified numerically.

## Conclusion

In this work we analyzed the well-known mass-conserved reaction-diffusion model for cell polarization of Otsuji et al. [14] as a representative of a model class from the perspective of pattern formation theory. The model belongs to a class that describes the dynamics of protein molecules that change from a slow diffusing membrane-bound state to a fast diffusing state in the cytosol and vice versa. We showed for this representative how such mass-conserved models comprising several equations can be mapped by a recently introduced method [41] to the single universal Cahn-Hilliard (CH) equation. Additionally, we present an extension to a general mass-conserved reaction- diffusion system of a similar form in S1 Appendix. Our perturbative reduction technique directly connects the parameters of the original cell polarization model to those of the CH equation. Because of this system-specific link between the polarization model and the CH equation, the analytical solution of the latter also become solutions of the original model.

Comparing numerical stationary solutions of the basic model, the CH model and analytical solutions of the CH model reveals a convincing agreement between all three approaches. In a comparison of the dynamics we also found coarsening behavior in large 2D systems. Near onset of cell polarization we find an almost perfect quantitative agreement between the polarization model and the CH equation while the solutions of the CH model still provide predictions on a qualitative level in even larger parameter ranges. The CH equation is the universal order parameter equation for mass-conserved reaction-diffusion systems near instabilities like that of the model of Otsuji et al., near so-called type II stabilities, cf. Ref. [3]. This also explains their different behavior compared to classical reaction-diffusion models leading to Turing patterns via a finite wavelength instability. While Turing models can often be reduced to the Ginzburg-Landau equation as a universal order parameter equation [3], the CH equation takes that role for mass-conserved reaction-diffusion models. A common underlying order parameter equation for different models of cell polarization explains why they often behave in a similar way in large parameter ranges. Hence, studying mass conserved models of cell polarization via a reduction to the CH model can help to identify and explain the universal and generic features cell polarization.

One of them is the instability of multi-peak solutions that (almost) always leads to a fully polar system. The inherent coarsening behaviour of the CH equation with typical scaling laws [38] ensures exactly this behavior. The initially growing pattern is unstable towards one with a larger wavelength. Thereby the system settles in the largest possible wavelength—which is the system length, corresponding to a single-peak solutions observed in 1D. However, a long lasting coarsening process from a solution with a large number of peaks might be not the desired way for a cell to reach a polar, i.e. single-peak state. In this case our analysis also allows the identification of the parameter region where a direct transition from the homogenous state to the polar state takes place without undergoing a complex coarsening process. On the other hand the instability of i.e. a double-peak solution towards a single peak enables the cell to always adapt their polarization pattern to the cell length using the generic feature of coarsening to its advantage. This adaptability of the polar zone to the cell length is crucial for biological tasks such as cell division or the transition to a moving cell.

Spontaneous polarization without an external gradient is another feature observed in several models for cell polarization [42]. In the CH model this would correspond to the onset of polarization with a positive control parameter. In this case the CH model develops into a polarized state for both the symmetric and the asymmetric case. Another important task in many cell polarization systems is the sustainability of the polar or single-peak state, i.e. the pattern has to persist even if the external stimulus is no longer present [18, 34]. In the CH model this would correspond to the bistability between the homogeneous and the polarized state in the asymmetric case. In this bistable range a temporal increase of the control parameter (above *ε* = 0) could push the system into the polarized state. If the control parameter then decreases again to subcritical values, the polarized state remains due to the hysteretic behavior in the bistable region. However, in this model this is only possible for asymmetric polarization (*γ*_3_ ≠ 0). To extend the sustainability also to symmetric polarization, a (intrinsic) subcritical bifurcation in the original model is necessary. In the picture of the Cahn-Hilliard equation this would lead to a change in the sign of the cubic nonlinearity *γ*_4_. To stabilize the system in this case, higher order nonlinearities have to be taken into account. The Cahn-Hilliard equation has to be extended up to (at least) quintic order which will provide further generic aspects of cell polarization in upcoming works.

## Materials and methods

### Simulation methods

We solve the cell polarization model in Eqs (1), (2) and (3) numerically by using a pseudo-spectral method. We calculate all spatial derivatives by transformation to a suitable function space depending on the boundary conditions. For periodic boundaries used here i.e. *u*|_*x*=0_ = *u*|_*x*=*L*_, *v*|_*x*=0_ = *v*|_*x*=*L*_, where *L* is the system length, we use a Fourier representation of the fields. For Figs 2 and 3 in the main text, we use a system length *L* = 800 and *N* = 512 modes in Fourier space. The initial condition is a step-like function of the form

$$\begin{array}{c} u\left(x\right)=A[\tanh(\frac{x-x_{l}}{\delta })-\tanh(\frac{x-x_{r}}{\delta })]-C, \end{array}$$ (42)

where we choose *C* such that $\int_{0}^{L}u\left(x\right)dx=0$ to fulfill the conservation law. We let this initial condition relax to a steady state. These steady state solutions are shown in Fig 2A and 2B and are also used to calculate the plateau values for different *ε* and *a*_2_ values in Figs 2B and 3B, 3C and 3D respectively (all references refer to the main text). Figs 2A and 3A show only one half of the system. The second half is axially symmetric and thus does not contain additional information. Note that due to this inherent symmetry of the profiles, the result for periodic boundary conditions with a system size *L* are equivalent to those with no-flux boundaries and the system size *L*/2.

For the simulations in 2D in Fig 4 we use *L*_*x*_ = *L*_*y*_ = 800 and *N*_*x*_ = *N*_*y*_ = 512, starting from random initial conditions.

### Calculation of the coarsening dynamics

The domain size *L*(*t*) in Fig 4B is determined via

$$\begin{array}{c} L\left(t\right)=2\pi \frac{\sum S(k_{i},t)dk}{\sum S\left(k_{i},t\right)k_{i}dk}, \end{array}$$ (43)

where *S*(*k*_*i*_, *t*) is the spherically averaged structure factor

$$\begin{array}{c} S\left(k_{i},t\right)=\left\langle \right|a_{k_{i}}{{|}^{2}\rangle }_{k_{i}}. \end{array}$$ (44)

$a_{k_{i}}$ are the coefficients of the two dimensional Fourier transform whereby ${\langle \rangle }_{k_{i}}$ denotes the radial average over all **k***_i_* with k*_i_* = |**k***_i_*|.

## Supporting information

S1 Appendix Derivation of CH equation from a cell polarization model. (PDF)

S2 Appendix Analytical calculation of plateau values. (PDF)
