# Supplementary material for: On system-spanning demixing properties of cell polarization
Source: PLoS One. 2019 Jun 21;14(6):e0218328. doi: 10.1371/journal.pone.0218328 (PMC6588261; doi:10.1371/journal.pone.0218328)
Supplement: S1 Appendix — (PDF) [file pone.0218328.s001.pdf]

On system-spanning demixing properties of cell polarization

Fabian Bergmann[1], Walter Zimmermann[1*],

1 Theoretische Physik I, Universität Bayreuth, 95440 Bayreuth, Germany

* walter.zimmermann@uni-bayreuth.de

S1 Appendix. Derivation of CH equation from a cell polarization model

We consider a model for a conserved reaction diffusion system with concentration fields $\tilde{u}$ and $\tilde{v}$ as described in the main text. The two coupled equations are

$$\partial_t \tilde{u} = D_u \nabla^2 \tilde{u} + f(\tilde{u}, \tilde{v})\,, \tag{1}$$

$$\partial_t \tilde{v} = D_v \nabla^2 \tilde{v} - f(\tilde{u}, \tilde{v})\,. \tag{2}$$

In the end we will apply our reduction scheme to the reaction term

$$f(\tilde{u}, \tilde{v}) = a_1 \left(\tilde{v} - \frac{\tilde{u} + \tilde{v}}{(1 + a_2(\tilde{u} + \tilde{v}))^2} \right), \tag{3}$$

while keeping the solution as general as possible. The homogeneous basic states (u_h, v_h) are determined by

$$f(u_h, v_h) = 0$$

and the conservation condition

$$N = \frac{1}{L} \int_0^L [u_h + v_h] dx = u_h + v_h.$$

In case of the reaction term as in Eq (3) the basic states are

$$u_h = \frac{a_2 N^2 (a_2 N + 2)}{(a_2 N + 1)^2}, \tag{4}$$

$$v_h = \frac{N}{(a_2 N + 1)^2}. \tag{5}$$

A linear stability analysis of the homogenous basic state leads to the growth rate σ for a small perturbation:

$$\sigma_\pm = \frac{1}{2} \left[\delta_1 \pm \sqrt{(f_u - f_v)^2 + 2q^2 \delta_2 + q^4 \delta_3^2} \right], \tag{6}$$

where

$$\delta_1 = -\left(D_u + D_v\right) q^2 + f_u - f_v\,,$$
$$\delta_2 = \left(f_u + f_v\right)\left(D_v - D_u\right)\,,$$
$$\delta_3 = D_v - D_u\,,$$

and

$$f_u = \frac{\partial f}{\partial \tilde{u}}\bigg|_{\tilde{u}=u_h,\tilde{v}=v_h} = \frac{a_1(a_2N-1)}{(a_2N+1)^3}\,, \tag{7}$$

$$f_v = \frac{\partial f}{\partial \tilde{v}}\bigg|_{\tilde{u}=u_h,\tilde{v}=v_h} = \frac{a_1a_2N(a_2^2N^2+3a_2N+4)}{(a_2N+1)^3}\,. \tag{8}$$

Note that the second equal sign only applies in the case of $f(\tilde{u},\tilde{v})$ as defined in Eq (3) Expanding the maximum growth rate for small values of q in the case $f_v > f_u$, we find

$$\sigma_+ = G_2q^2 - G_4q^4 + \mathcal{O}(q^6) \tag{9}$$

with

$$G_2 = \frac{D_vf_u - D_uf_v}{f_v - f_u}\,,$$
$$G_4 = \frac{(D_u-D_v)^2f_uf_v}{(f_v-f_u)^3}\,.$$

Note that for $f(\tilde{u},\tilde{v})$ given by Eq (3) we have $f_v - f_u = a_1$. This means for positive a_1, the inequality $f_v > f_u$ is fulfilled. Eqs (1), (2) can therefore be written in a compact form as follows

$$\partial_t\mathbf{w} = \mathcal{L}\mathbf{w} + \mathbf{N}\,, \tag{10}$$

with $\mathbf{w} = (u = \tilde{u} - u_h, v = \tilde{v} - v_h)$. Thereby we expand the reaction term $f(\tilde{u},\tilde{v})$ in a Taylor series around the homogenous state (u_h, v_h):

$$\begin{aligned} f(\tilde{u},\tilde{v}) \approx & f(u_h,v_h) + f_uu + f_vv + \\ & \frac{1}{2}f_{uu}u^2 + f_{uv}uv + \frac{1}{2}f_{vv}v^2 + \ldots\,, \end{aligned} \tag{11}$$

where u, v are the deviations from the homogeneous state and $f_{uu} = \partial_u^2 f|_{u=u_h,v=v_h}$ etc. This leads to the linear part $\mathcal{L}$ with

$$\mathcal{L} = \begin{pmatrix} D_u\partial_x^2 + f_u & f_v \\ -f_u & D_v\partial_x^2 - f_v \end{pmatrix}\,. \tag{12}$$

Applying the scalings and expansions of the perturbative reduction scheme as described in the main text (see Eqs (20), (21), (22), (23)) and separating the resulting equation with respect to the orders of ε leads to a hierarchy of equations in Eqs (24)-(28). The time scaling T_3 will become clear during the calculation. At the order $\sqrt{\varepsilon}$ of this hierarchy we find the equation

$$\mathcal{L}_0\mathbf{w}_1 = 0. \tag{13}$$

This is an equation for an eigenvector corresponding to the eigenvalue $\sigma = 0$ and can be solved by

$$\mathbf{w}_1 = \tilde{A}(X, T_3, T)\begin{pmatrix} f_v \\ -f_u \end{pmatrix}\,, \tag{14}$$

whereby $(f_v, -f_u)^T$ is the respective eigenvector. The prefactor $\tilde{A}$ cannot be calculated by this linear equation. Instead we have to look at the equations of higher orders in the

hierarchy (see Eqs (24)-(28)) to find an equation that determines $\tilde{A}$ and thereby $\mathbf{w} \approx \mathbf{w_1}$ close to $\varepsilon = 0$. At the order ε we find an equation for $\mathbf{w}_2$:

$$\mathcal{L}_0 \mathbf{w}_2 = -N_2 \begin{pmatrix} 1 \\ -1 \end{pmatrix} , \tag{15}$$

with

$$N_2 = \frac{1}{2} f_{uu} u_1^2 + f_{uv} u_1 v_1 + \frac{1}{2} f_{vv} v_1^2 \tag{16}$$

containing all the nonlinear terms of order ε. Note that due to the general form of $\mathbf{w}_1$ (see Eq (14)), N_2 only contains terms $\propto \tilde{A}^2$. Since the left hand side of Eq (15) and Eq (13) are the same, potential secular terms on the right hand side have to vanish. This solvability condition is also called the Fredholm alternative. In our case this means that Eq (15) only has a solution if the right hand side is perpendicular to the left eigenvector of $\mathcal{L}_0$. This left eigenvector is

$$\mathbf{z} = \begin{pmatrix} 1 \\ 1 \end{pmatrix} . \tag{17}$$

If we project the right hand side of Eq (15) onto the left eigenvector, we find the Fredholm alternative always fulfilled (because $\mathbf{z}$ is perpendicular to $(1, -1)$) and no secular terms appear in this order. Therefore we can directly solve Eq (15) for $\mathbf{w}_2$. Since we have only one equation to determine u_2 and v_2, we have an additional degree of freedom. We choose this in a way that the solution at order ε is

$$\mathbf{w}_2 = \frac{1}{f_v - f_u} \left(N_2 \begin{pmatrix} 1 \\ -1 \end{pmatrix} + \tilde{B}(X, T_3, T) \begin{pmatrix} f_v \\ -f_u \end{pmatrix} \right) , \tag{18}$$

with $\tilde{B}(X, T_3, T)$ reflecting the additional degree of freedom. At order $\varepsilon^{3/2}$ we find

$$\mathcal{L}_0 \mathbf{w}_3 = -\mathcal{L}_1 \partial_X^2 \mathbf{w_1} - N_3 \begin{pmatrix} 1 \\ -1 \end{pmatrix} , \tag{19}$$

with

$$\mathcal{L}_1 = \begin{pmatrix} D_u & 0 \\ 0 & D_{v,c} \end{pmatrix} \tag{20}$$

and

$$\begin{aligned} N_3 = &\frac{1}{6} f_{uuu} u_1^3 + \frac{1}{2} f_{uuv} u_1^2 v_1 + \frac{1}{2} f_{uvv} u_1 v_1^2 + \frac{1}{2} f_{vvv} v_1^3 \\ &+ f_{uu} u_1 u_2 + f_{uv}(u_1 v_2 + u_2 v_1) + f_{vv} v_1 v_2 \, . \end{aligned} \tag{21}$$

Due to the solutions $\mathbf{w}_1$ and $\mathbf{w}_2$ contains contributions $\propto \tilde{A}^3$ and $\propto \tilde{A}\tilde{B}$. Since $D_u f_v - D_{v,c} f_u = 0$, the Fredholm alternative is again automatically fulfilled in this order. Solving Eq (19) leads to

$$\mathbf{w}_3 = \frac{D_u f_v \partial_X^2 \tilde{A} + N_3}{f_v - f_u} \begin{pmatrix} 1 \\ -1 \end{pmatrix} + \frac{\tilde{C}(X, T_3, T)}{f_v - f_u} \begin{pmatrix} f_v \\ -f_u \end{pmatrix} . \tag{22}$$

At order ε^2 the application of the Fredholm alternative on the right hand side of

$$\mathcal{L}_0 \mathbf{w}_4 = \partial_{T_3} \mathbf{w}_1 - \mathcal{L}_1 \partial_X^2 \mathbf{w}_2 - N_4 \begin{pmatrix} 1 \\ -1 \end{pmatrix} \tag{23}$$

is not automatically fulfilled. Instead the projection of the right hand side to the left eigenvector (17) leads to

$$\partial_{T_3}\tilde{A} = \frac{D_{v,c} - D_u}{(f_v - f_u)^2}\partial_X^2 N_2\,. \tag{24}$$

Note that in this order of ε the time scale T_3 is necessary to fulfill the Fredholm condition. Otherwise the Fredholm alternative would demand $\partial_X^2 N_2 = 0$ which would render Eq (23) unsolvable. Solving for $\mathbf{w}_4$ in Eq (23) is possible but turns out to be not necessary to determine a evolution equation for $\tilde{A}$. But since Eq (24) does not contain any amplitude-restricting term, we still have to go to a higher order in ε to find an equation for the amplitude $\tilde{A}$. Indeed, the solvability condition (Fredholm alternative) in order $\varepsilon^{5/2}$ provides the evolution equation for $\tilde{A}$:

$$\begin{aligned}&(f_v - f_u)\partial_T\tilde{A} + \partial_{T_3}\tilde{B} = \\ &\qquad - D_{v,c} f_u \partial_X^2\tilde{A} + \frac{D_u - D_{v,c}}{f_v - f_u}\partial_X^2\left(D_u f_v \partial_X^2 \tilde{A} + N_3\right).\end{aligned} \tag{25}$$

In this equation we separate the terms exclusively containing $\tilde{A}$ but not $\tilde{B}$ (contained in N_3). This goes back to original separation of scales. Close to $\varepsilon = 0$ only the amplitude $\tilde{A}$, but not $\tilde{B}$ is relevant for $\mathbf{w}$. This means that the amplitude $\tilde{A}$ alone should determine the solution of u and v close to the onset of cell polarization. We now assume Eq (25) to hold for any (small) value of ε. Since we know that for $\varepsilon \to 0$ only the value of $\tilde{A}$ is relevant, Eq (25) has to be solvable for arbitrary values of $\tilde{B}$ – especially for the value $\tilde{B} = 0$. Therefore Eq (25) can be split into an equation for $\partial_T A$, whose right hand side contains only terms that solely depend on $\tilde{A}$ and a separate equation for $\partial_{T_3} B$. Inserting the values for the nonlinearities derived from Eq (3) we find

$$\begin{aligned}N_2 &= -\frac{a_1^3 a_2(a_2 N - 2)}{(a_2 N + 1)^4}\tilde{A}^2\,,\\ N_3 &= \frac{a_1^4 a_2 (a_2 N - 3)}{(a_2 N + 1)^5}\tilde{A}^3 + \frac{2a_1^2 a_2 (2 + a_2 N - a_2^2 N^2)}{(a_2 N + 1)^5}\tilde{A}\tilde{B}\,.\end{aligned}$$

Therefore we finally end up with

$$\partial_{T_3}\tilde{A} = -\frac{D_u a_1 a_2 (a_2 N - 2)}{a_2^2 N^2 - 1}\partial_X^2 \tilde{A}^2\,, \tag{26}$$

$$\begin{aligned}\partial_T\tilde{A} = -\partial_X^2\Bigg(&\frac{D_u a_2 N (a_2^2 N^2 + 3a_2 N + 4)}{(a_2 N + 1)^3}\tilde{A}\\ &+ \frac{D_u^2 a_2 N (a_2^2 N^2 + 3 a_2 N + 4)}{a_1 (a_2 N - 1)}\partial_X^2\tilde{A}\\ &+ \frac{D_u a_1^2 a_2^2 (a_2 N - 3)}{(a_2 N + 1)^2 (a_2 N - 1)}\tilde{A}^3\Bigg)\end{aligned} \tag{27}$$

as the order parameter equation for the reaction term Eq (3). We now return from the scales X, T_3 and T to the original ones (x and t) while also substituting $\tilde{A} = A/\sqrt{\varepsilon}$:

$$\begin{aligned}\partial_t A &= \varepsilon^{3/2}\partial_{T_3} A + \varepsilon^2 \partial_T A\\ &= \varepsilon^2 \partial_{T_3}\tilde{A} + \varepsilon^{5/2}\partial_T \tilde{A}\\ &= -\partial_x^2\left(\gamma_1 A + \gamma_2 \partial_x^2 A - \gamma_3 A^2 - \gamma_4 A^3\right)\,.\end{aligned} \tag{28}$$

Note that the order parameter equation for an arbitrary reaction term $f(\tilde{u}, \tilde{v}$ always takes this form. Only the coefficients γ_i are system specific. For the reaction term in Eq. (3) these are defined in the main text.
