# Supplementary material for: On system-spanning demixing properties of cell polarization
Source: PLoS One. 2019 Jun 21;14(6):e0218328. doi: 10.1371/journal.pone.0218328 (PMC6588261; doi:10.1371/journal.pone.0218328)
Supplement: S2 Appendix — (PDF) [file pone.0218328.s002.pdf]

# On system-spanning demixing properties of cell polarization

Fabian Bergmann[1], Walter Zimmermann[1*],

**1** Theoretische Physik I, Universität Bayreuth, 95440 Bayreuth, Germany

* walter.zimmermann@uni-bayreuth.de

### S2 Appendix. Analytical calculation of plateau values

The CH equation

$$\partial_t A = -\partial_x^2 \left( \gamma_1 \varepsilon A + \gamma_2 \partial_x^2 A - \gamma_3 A^2 - \gamma_4 A^3 \right) \tag{1}$$

can be derived from the functional

$$F = -\int_0^L \frac{\gamma_1 \varepsilon}{2} A^2 - \frac{\gamma_2}{2} (\partial_x A)^2 - \frac{\gamma_3}{3} A^3 - \frac{\gamma_4}{4} A^4 dx \tag{2}$$

via

$$\partial_t A = \partial_x^2 \frac{\delta F}{\delta A} \,. \tag{3}$$

To determine the plateau values for $A$, we look for the extremal values of $F$. Due to the conservation condition we cannot simply calculate the extrema of the integrant, but have to determine $F$. For reasons of simplicity we neglect the interface energy term $\gamma_2(\partial_x A)^2$ in the functional. We assume a plateau solution with an upper plateau $A_+$ over a length $L_+$ and a lower plateau over the length $L_-$. The conservation condition then leads to

$$\begin{aligned} &A_+ L_+ + A_- L_- = 0 \,, \\ &A_- = -\frac{L_+}{L_-} A_- = -\left( \frac{L}{L_-} - 1 \right) A_+ = -(\lambda - 1) A_+ \,, \end{aligned} \tag{4}$$

with $L = L_+ + L_-$ and $\lambda = L/L_-$. Therefore the value of the functional becomes

$$\begin{aligned} f = \frac{F}{L} &= -\left( \frac{\gamma_1 \varepsilon}{2} A_+^2 - \frac{\gamma_3}{3} A_+^3 - \frac{\gamma_4}{4} A_+^4 \right) \frac{L_+}{L} \\ &\quad - \left( \frac{\gamma_1 \varepsilon}{2} A_-^2 - \frac{\gamma_3}{3} A_-^3 - \frac{\gamma_4}{4} A_-^4 \right) \frac{L_-}{L} \\ &= -(\lambda - 1) \Bigg( \frac{\gamma_1 \varepsilon}{2} A_+^2 - \frac{\gamma_3}{3} (2 - \lambda) A_+^3 \\ &\qquad - \frac{\gamma_4}{4} (\lambda^2 - 3\lambda + 3) A_+^4 \Bigg) \,. \end{aligned} \tag{5}$$

The extrema of this functional with respect to $A_+$ and $\lambda$ are determined by the following equations:

$$\frac{\partial f}{\partial A_+} = \gamma_1 \varepsilon - \gamma_3 (2 - \lambda) A_+ - \gamma_4 (\lambda^2 - 3\lambda + 3) A_+^2 = 0 \,, \tag{6}$$

$$\frac{\partial f}{\partial \lambda} = \frac{\gamma_1 \varepsilon}{2} - \frac{\gamma_3}{3} (3 - 2\lambda) A_+ - \frac{\gamma_4}{4} (3\lambda^2 - 8\lambda + 6) A_+^2 = 0 \,. \tag{7}$$

Solving these coupled equations finally leads to

$$A_{\pm} = \frac{-\gamma_3 \pm \sqrt{3\gamma_3^2 + 9\gamma_1\gamma_4\varepsilon}}{3\gamma_4}\,. \tag{8}$$

This equation allows to calculate the critical point above which to expect a nonzero amplitude. The amplitude according to Eq (8) is real-valued for $\varepsilon > -\gamma_3^2/(3\gamma_1\gamma_4)$. But due to the conservation law, the value of the lower plateau has to be negative while the value of the higher plateau has to be positive. This condition leads to

$$\varepsilon > -\frac{2\gamma_3^2}{9\gamma_1\gamma_4}\,. \tag{9}$$

In simulations the control parameter value where to find cell polarization will be slightly higher than the one analytically calculated due to the surface energy term that was neglected in the calculation.
